# The relationship between indoleamine 2,3-dioxygenase activity and post-stroke cognitive impairment

**DOI:** 10.1186/1742-2094-8-17

**Published:** 2011-02-16

**Authors:** Allison B Gold, Nathan Herrmann, Walter Swardfager, Sandra E Black, Richard I Aviv, Gayla Tennen, Alexander Kiss, Krista L Lanctôt

**Affiliations:** 1 Neuropsychopharmacology Research Group, Sunnybrook Health Sciences Centre, Toronto, Ontario, Canada; 2 Brain Sciences Research Program, Sunnybrook Health Sciences Centre, Toronto, Ontario, Canada; 3 Heart and Stroke Foundation Centre for Stroke Recovery, Toronto, Ontario, Canada; 4 Department of Pharmacology/Toxicology, University of Toronto, Toronto, Ontario, Canada; 5 Department of Medicine (Neurology), University of Toronto, Toronto, Ontario, Canada; 6 Department of Medical Imaging (Neuroradiology), University of Toronto, Toronto, Ontario, Canada; 7 Institute for Clinical Evaluative Sciences, Toronto, Ontario, Canada; 8 Department of Psychiatry, University of Toronto, Ontario, Canada

## Abstract

**Background:**

Activation of indoleamine 2,3-dioxygenase (IDO) and higher concentrations of several kynurenine metabolites have been observed post-stroke, where they have been associated with increased mortality. While lower tryptophan or a higher ratio of kynurenine/tryptophan (K/T) in peripheral blood have been associated with dementia and the severity of cognitive symptoms in Alzheimer's disease, the association between K/T ratios and post-stroke cognitive impairment (PSCI) has not been investigated.

**Methods:**

Patients were recruited from the acute stroke unit of a general hospital within 1 month post-stroke. Assessments included the Standardized Mini-Mental State Examination (sMMSE) for cognition, the National Institutes of Health Stroke Scale (NIHSS) for stroke severity, and the Center for Epidemiological Studies-Depression Scale (CES-D) for depressive symptoms. Tryptophan and kynurenine concentrations were determined by high-performance liquid chromatography.

**Results:**

A total of 41 patients with ischemic stroke ([mean ± SD] age 72.3 ± 12.2 years, 53.7% male, sMMSE 25.6 ± 4.1, NIHSS 7.27 ± 5.55) were recruited. Higher K/T ratios were associated with lower post-stroke global cognition (i.e. sMMSE scores; β = -.327, P = .037). A backward stepwise elimination linear regression (F_1,40_=6.15, P=.005, adjusted R^2^=.205) showed that the highest K/T ratio tertile (β = -.412, P = .006) predicted lower sMMSE scores, controlling for age (β = -.253, p = .081), with NIHSS (β = -.027, P = 0.859), and lesion volume (β = -.066, P = 0.659) removed from the model. In receiver operating characteristic analysis, a K/T ratio of 78.3 μmol/mmol (top tertile) predicted significant cognitive impairment (sMMSE score ≤ 24) with 67% sensitivity and 86% specificity (area under the curve = 0.730, p = .022).

**Conclusions:**

These data suggest an inflammatory response characterized by IDO activation may be relevant to the development of PSCI. Since the neuroactivity of kynurenine metabolites may be amenable to pharmacotherapeutic intervention, the K/T ratio may be a clinically important biomarker.

## Background

Stroke affects 15 million individuals annually world-wide, and the risk of having a stroke more than doubles each decade after the age of 55[1]. Most stroke survivors live with residual impairments that diminish independence and quality of life[2]. For older patients with ischemic stroke, post-stroke cognitive impairment (PSCI) is particularly important and frequent, occurring in approximately one third of all patients[3] and having a significant negative impact on rehabilitation outcomes[4], quality of life[5] and risk of dementia[6]. A recent meta-analysis has confirmed several risk factors, including previous symptomatic stroke, previous asymptomatic stroke seen on imaging, multiple stroke lesions, aphasia, stroke severity, and stroke location, are associated with PSCI as defined by Mini-Mental State Examination (MMSE) scores less than 24, Diagnostic and Statistical Manual of Mental Disorders IV (DSM-IV) or International Classification of Disease-10 (ICD-10) criteria, within 1 year after stroke[7]. The prevalence of post-stroke MMSE scores less than 24, indicative of significant cognitive impairment, was much higher than the prevalence of dementia diagnosed by standard criteria, which included the DSM-IV or ICD-10[7]. The majority of these and other known risk factors for PSCI including older age, lower level of education, family history of dementia [8] are not readily amenable to treatment. Therefore, there is considerable need to identify pathophysiological mechanisms that may contribute to PSCI.

The systemic inflammatory response to acute ischemic stroke involves increases in several pro-inflammatory cytokines and C-reactive protein (CRP)[9-11], which have also been associated with the development of cognitive deficits and dementia in aging populations[12]. We have recently demonstrated a relationship between PSCI and two inflammatory biomarkers, CRP and interleukin-6 (IL-6)[13]. Pro-inflammatory cytokines can activate the indoleamine-2,3-dioxygenase (IDO) enzyme, leading to the depletion of tryptophan (TRP) and the production of kynurenine, increasing the kynurenine/tryptophan (K/T) ratio in peripheral blood, which acts as a clinical measure of IDO activity[14]. Elevations in the K/T ratio and in the concentrations of several kynurenine metabolites have been observed post-stroke, where they have been associated with mortality[15]. While lower peripheral blood tryptophan concentrations or higher K/T ratios have been associated with dementia and the severity of cognitive symptoms in Alzheimer's disease[16,17], the association between K/T ratios and PSCI has not been investigated.

Tryptophan metabolites along the kynurenine pathway can produce excitatory and oxidative neurotoxicity, but also protect neurons from inflammatory damage and attenuate excitatory neurotoxicity via NMDA receptor antagonism[18-23]. Therefore, it is of interest to determine whether kynurenine production might be associated with clinical cognitive outcomes, and if so, at which concentrations.

The purpose of this study was to test the hypothesis that IDO activation is associated with the presence of cognitive symptoms post-stroke. We measured plasma concentrations of kynurenine and tryptophan in acute ischemic stroke patients and explored the relationship between the K/T ratio and cognitive deficits, as measured by the MMSE, a commonly used cognitive screening instrument[24].

## Methods

### Study design

This cross-sectional observational study recruited participants admitted to an acute care regional stroke centre within 30 days of an ischemic stroke. The study was approved by local research ethics boards, and all participants provided written informed consent.

### Recruitment

Consecutive patients meeting the National Institute of Neurological and Communicative Disorders and Stroke (NINCDS)[25] and World Health Organization Multinational Monitoring of Trends and Determinants in Cardiovascular Disease (WHO-MONICA)[26] criteria for stroke were invited to participate in this study. Acute ischemic infarcts were verified from computed tomography (CT) or magnetic resonance imaging (MRI) reports performed at the time of admission in all patients. Inclusion criteria also required participants to speak and understand English. Exclusion criteria were: pre-stroke diagnosis of dementia or significant cognitive impairment, primary hemorrhagic stroke (which have a different etiology and clinical course), decreased level of consciousness, severe aphasia or dysarthria (which would interfere with the ability to complete the study assessments), significant acute medical illness (which would be associated with significant inflammatory burden of its own; e.g. infection, autoimmune disease, cancer), significant acute neurological illness other than stroke, and the presence of a premorbid axis I psychiatric disorder. A history of major depression was permitted, and controlled for in statistical analyses as needed.

### Assessments

After obtaining written informed consent, the Mini-Mental State Examination (MMSE)[24] was administered. The MMSE was selected as a cognitive screening instrument because it is a brief, widely used, and validated scale in acute care settings[27]. Although not sensitive to subtle cognitive impairment, scores on the MMSE correlate strongly with the more thorough yet lengthy CAMCOG (cognitive and self-contained part of the Cambridge Examination for Mental Disorders of the Elderly) in a stroke rehabilitation setting[28]. Given the exploratory nature of this study and the potentially low tolerability of a longer assessment in an acute stroke setting, a brief yet validated and clinically meaningful cognitive instrument was selected. MMSE scores were adjusted according to physical ability post-stroke as recommended by the Standardized Mini Mental State Examination (sMMSE)[29].

Stroke severity was assessed using the National Institutes of Health Stroke Scale (NIHSS)[30], either as completed by clinicians at the time of patient admission and obtained through chart review, or extracted from the chart information using a standardized method as has been done before [31]. Depressive symptoms were assessed using the Center for Epidemiological Studies-Depression Scale (CES-D)[32]. Information regarding demographic characteristics and medical history was collected via chart review and consultation with participants.

### Blood sampling and plasma analyses

Fasting blood was collected via venipuncture in EDTA (ethylenediaminetetraacetic acid) BD Vacutainer ® (New Jersey, USA) tubes at 7:30 am ± 30 minutes on the morning after the clinical assessments were conducted. Blood samples were centrifuged at 1000 × *g *for 10 min at 4°C and plasma was separated and stored at -80°C until the time of assay. Tryptophan and kynurenine concentrations were determined by high-performance liquid chromatography (HPLC), as described elsewhere[33,34]. Tryptophan was measured by isocratic reverse phase HPLC without derivatization and fluorescence detection. For kynurenine, an equal volume of 3% perchloric acid was used for protein precipitation. After centrifugation, the concentration of _L_-kynurenine in the supernatant was measured by HPLC with UV detection at 258 nm. The mobile phase consisted of 9% acetonitrile in 0.05 M potassium phosphate mono basic, pumped through a reverse phase 5 μm ODS column, 250 mm × 4.6 mm (Symmetry; Waters Corporation, Milford, Massachusetts, United States of America). Biochemical assays were performed blinded to all clinical information.

Lesion characteristics were determined from CT scans obtained without a contrast agent on a General Electric LightSpeed VCT series scanner (General Electric Healthcare, Waukesha, WI). Ischemic lesions were manually traced on these images using Medical Image Processing, Analysis, and Visualization (MIPAV; National Institutes of Health, Bethesda, MD). Stroke volume was calculated using a slice-by-slice planimetric methodology employing these manual lesion tracings.

### Statistical Analysis

Continuous measures were summarized using means and standard deviations whereas categorical measures were summarized using percentages. Tryptophan and kynurenine were determined by mass and converted to molar units. Their quotient was multiplied by 1000 to obtain the K/T ratio in units of μmol/mmol. The naturally skewed nature of the data resulted in absolute values of kurtosis and skewedness being greater than 2 SD of the error, so K/T values were log transformed to obtain a normal distribution. All statistical analyses were performed using transformed values or tertiles.

For initial descriptive analyses, the relationship between sMMSE scores and K/T ratio, as well as possible covariates such as age, gender, stroke severity, time since stroke, level of education, cumulative burden of cerebrovascular risk factors (i.e. the number of vascular risk factors including diabetes, smoking, hypertension, hyperlipidemia, and obesity summed), and lesion volume were analyzed with bivariate Pearson correlations or analyses of variance (ANOVA) as appropriate.

To test our hypothesis, a backward elimination multiple linear regression model was used to examine the association between K/T ratios and sMMSE scores as continuous variables with age, stroke severity, and lesion volume entered into the initial model. In addition, to determine if an elevated K/T ratio predicted poorer sMMSE scores, patients were divided into tertiles based on the K/T ratio, and a backward elimination multiple linear regression analysis with patients dichotomized by those in the top tertile vs. others was used. A removal criterion of P > .1 was used in backward regression models. Finally, a receiver operating characteristic (ROC) analysis was performed to determine a K/T ratio optimum (sensitivity vs. specificity) that predicts significant cognitive impairment (sMMSE score ≤24) [7].

Given the strong commonly observed relationships between depressive symptoms, inflammatory activation[35] and cognitive impairment[36,37], the impact of depressive symptoms or a history of depression on the observed relationship between K/T ratio top tertile and sMMSE was explored by adding CES-D scores or history of depression into the final regression model. Similarly, given the observed relationship between vascular risk factors and cognitive impairment[38], the number of cerebrovascular risk factors was added to the final regression model. Time since stroke, gender, and level of education were also explored as covariates.

All patient information was de-identified for statistical analyses using SPSS statistical software (version 17; SPSS Inc., Chicago, Illinois).

### Sample size and study power calculation

A sample size of 39 subjects achieves a power of 80% to detect an association between the K/T ratio and sMMSE scores with an effect size (f^2^) of 0.35 given a two-tailed significance level of 0.05. A sample size of  at least 41 subjects allows adjustment with up to 3 additional covariates.

## Results

A total of 41 patients (mean ± SD age 72.3 ± 12.2 years, 53.7% male) were recruited. Tryptophan and kynurenine chromatograms appeared as previously described[33]. The mean K/T ratio was 67.40 ± 42.46 μmol/mmol. Scores on the sMMSE ranged from 13 to 30, with 29.3% of the sample having evidence of significant cognitive impairment (sMMSE ≤ 24). Table 1 reports the results of correlations with sMMSE scores and K/T ratios, as well as between sMMSE scores and demographic and lesion characteristics. In addition to K/T ratio, time since stroke and level of education were associated with sMMSE scores, with a trend for age to be significantly associated (Table 1). No association between lesion volume and sMMSE scores was found.

**Table 1:** Clinical characteristics and correlations with cognitive impairment (n = 41)

	MEAN ± SD OR %	RANGE	r- OR F* VALUE	P-VALUE^†^
Age	72.3 ± 12.2 years	39-91 years	-0.27	0.08
Gender	53.7% male		1.83	0.18
MMSE	25.6 ± 4.1	13-30	-	-
NIHSS	7.27 ± 5.55	0-21	-0.08	0.63
CES-D	13.8 ± 12.8	0-48	-0.09	0.59
History of Depression	7.3%		0.35	0.56
Living Alone	24.4%		0.06	0.81
Level of Education > high school	46.3%		6.64	0.01
Time Since Stroke	9.17 ± 5.03 days	4-26 days	-0.34	0.03
Cerebrovascular Risk Factors				
Hypertension	85.4%		4.53	0.04
Diabetes	26.8%		0.21	0.65
Hyperlipidemia	34.1%		0.41	0.52
Obesity (BMI ≥ 30kg/m^2^)	31.7%		2.17	0.15
Smoking	22.0%		0.10	0.75
Total number risk factors	2.15 ± 1.28	0-5	-0.21	0.19
Lesion Volume (cm^3^)	31.72 ± 55.09	0.250 -287.58	-0.05	0.76
K/T ratio ^‡^	67.40 ± 42.46	14.04-203.75	-0.33	0.04

Abbreviations: MMSE, Mini-Mental State Examination; NIHSS, National Institutes of Health Stroke Scale; CES-D, Center for Epidemiological Studies-Depression Scale; BMI, body mass index; K/T ratio, Kynurenine/Tryptophan ratio

*r or F values and their corresponding ^†^p-values reflect the results of bivariate Pearson correlations with sMMSE scores for continuous variables and ANOVA between sMMSE scores -for categorical variables;^‡ ^values reflect the mean ± SD of the detectable samples in each group.

**Figure 1:**
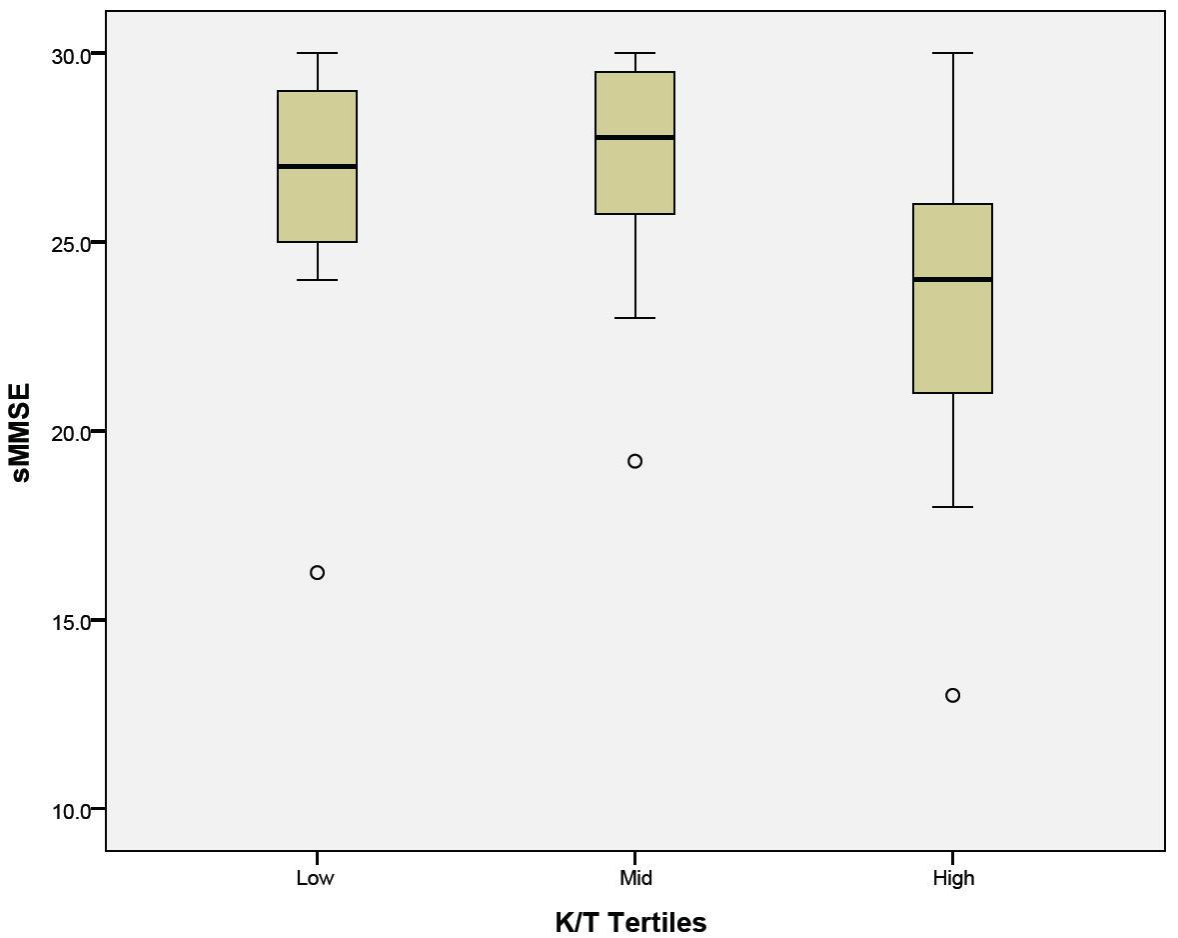
**Box and whisker plot representing the relationship between standadized Mini-Mental State Examination (sMMSE) scores and K/T tertiles, represented as low (mean ± SD = 29.07 ± 7.31), middle (mean ± SD = 58.39 ± 10.86), high (mean ± SD = 116.12 ± 39.44)**. The black horizontal line in each box represents the median. Outliers (circles) are recorded individually.

The bivariate correlation between sMMSE scores and the K/T ratio persisted (β = -.327, P = .037) in a backward stepwise elimination linear regression where age, lesion volume, and stroke severity were removed from the model (adjusted R^2 ^= .084). When patients were dichotomized by those in the highest K/T ratio tertile versus others, being in the top tertile emerged as a significant predictor of sMMSE scores (β = -.412, P = .006) when controlling for age (β = -.253, P = .081) with stroke severity (β=-.027, P=0.859) and lesion volume (β=-.066, P=0.659) removed from the final model (adjusted R^2 ^= .205, F_1,40 _= 6.15, p = .005; Table 2). When exploring gender, time since stroke, level of education, number of cerebrovascular risk factors, history of depression, or CES-D scores as covariates, the association between the K/T ratio top tertile and sMMSE scores remained significant (P < .018). A plot depicting the relationship between the K/T tertiles and sMMSE scores is provided in Figure 1.

**Table 2:** Linear regression model predicting sMMSE scores*

	B	Std. Error	β	P
K/T top tertile	-3.61	1.28	-.410	.008*
NIHSS	-.020	.111	-.027	.859
Age	-.085	.049	-.254	.088
Lesion Volume	-4.95E-6	.000	-.066	.659

*Starting block with all covariates entered (overall adjusted R^2 ^= .167, F_1, 40 _= 3.01, p = .031). After backward elimination (p > .1), K/T ratio top tertile (β = -.412, p = .006) and age (β = -.253, P = .081) remained in the model (overall  F_1, 40 _= 6.15, p = .005, adjusted R^2 ^= .205).

**Figure 2:**
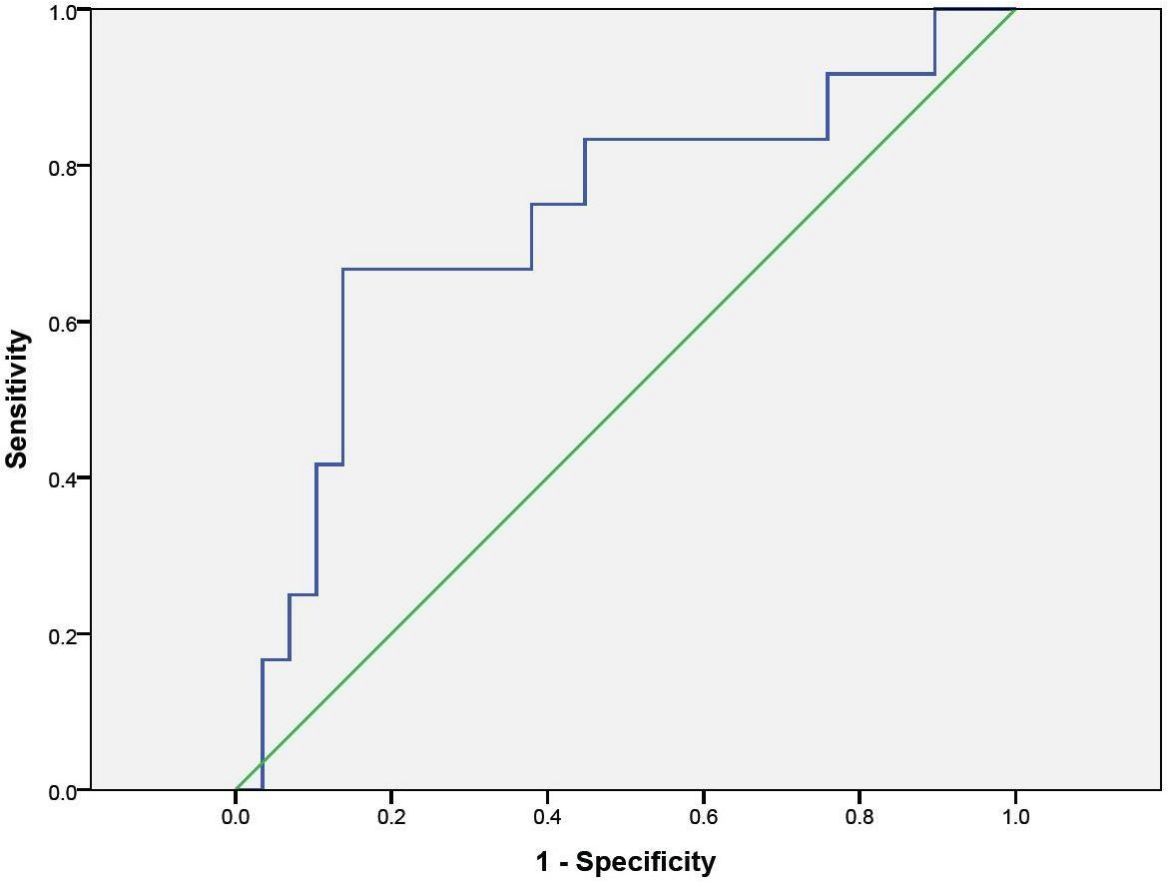
**Optimal K/T to detect significant cognitive impairment**. Receiver Operating Characteristic Curve. A K/T ratio of 78.3 μmol/mmol predicted significant cognitive impairment (sMMSE score ≤ 24) with 67% sensitivity and 86% specificity. Area under the curve = .730[.548, .912]; Std Error = .093, p = .022.

In an ROC curve, a K/T ratio of 78.3 μmol/mmol predicted the presence of an sMMSE score ≤ 24 with 67% sensitivity and 86% specificity (Figure 2). The area under the curve was .730, indicating that 73.0% of the subjects will have a K/T ratio that correctly ranks their likelihood of having an sMMSE score ≤ 24.

## Discussion

This study demonstrates an association between elevated K/T ratios and the extent of cognitive impairment among acute ischemic stroke patients. Age, stroke severity and lesion volume did not significantly modify this relationship suggesting that the clinical importance of the K/T ratio may extend beyond their relationships with these established risk factors for PSCI. The association between the K/T ratio and cognition following acute stroke was sufficiently robust to be detected with the MMSE, a practical and clinically meaningful cognitive screening instrument.

Inflammatory activation is a key process in the ischemic cascade that leads to secondary brain damage[39]. We have previously reported that plasma CRP concentrations were able to explain 20% of the variance in MMSE scores among acute stroke patients and that plasma IL-6 concentrations also predicted MMSE scores[13]. Interestingly, high plasma concentrations of the pleiotrophic cytokine, IL-6, have been associated with a greater lesion volume[40], however lesion volume did not predict sMMSE scores in our population. Pro-inflammatory cytokines induce the expression of IDO which catalyzes the committal and rate-limiting step in the production of kynurenine from tryptophan. In the present study, having a K/T ratio in the top tertile explained 21% of the variance in MMSE scores.

An elevated K/T ratio has been associated with inflammatory and neurodegenerative conditions and with clinically important outcomes, including mortality in the very old[41], poorer cognitive function in those with Alzheimer's disease[17], and the severity of depressive symptoms in those receiving interferon therapy[42] or in those with cardiovascular disease[43]. The K/T ratio and poorer MMSE scores may be related to the burden of neurotoxic or neuroactive metabolites such as quinolinic acid (QUIN), an NMDA receptor agonist and excitatory neurotoxin, 3-hydroxyanthranilic acid, an oxidative neurotoxin, or kynurenic acid (KYNA), an antagonist at both NMDA and α7 cholinergic receptors. The production of these neuroactive metabolites has been previously associated with depression[44,45], cognitive impairment, and deficits in learning, retrieval, and long-term memory[40,46]. For instance, the concentration of KYNA is elevated in the caudate nucleus and putamen of Alzheimer disease patients[47] and it has been suggested that NMDA or α7 cholinergic inhibition by KYNA might impair learning and memory[48]. In a study by Widner et al., Alzheimer's disease patients showed significantly higher peripheral blood K/T ratios as compared to age-matched controls and Alzheimer's disease patients with the lowest MMSE scores had significantly higher K/T ratios than those with the highest MMSE scores[17]. A second study by Gulaj et al. did not find a correlation between K/T ratios and MMSE scores; however they observed an association between the KYNA/KYN ratio and MMSE scores, suggesting a possible protective effect of KYNA relative to the production of kynurenine and other metabolites[16]. This effect may be attributable to opposing effects of KYNA and quinolinic acid at NMDA or α7 nicotinic cholinergic receptors. The association between increased concentrations of kynurenine metabolites, especially KYNA and 3-hydroxyanthranilic acid, and mortality observed post-stroke[15] suggests the potential clinical importance of these metabolites in this population.

Identification of the K/T ratio as a PSCI biomarker may represent an important step in improving stroke outcomes because the kynurenine metabolites might be amenable to pharmacological manipulation[23]. In the central nervous system, kynurenine aminotransferase II, expressed primarily in astrocytes, is responsible for KYNA synthesis while kynurenine 3-monooxygenase (KMO) produces the 3-hydroxykynurenine metabolite that gives rise to QUIN, predominantly from the microglia[49]. In animal models of cerebral ischemia, administration of kynurenine sulfate exerts neuroprotective effects and notably increases cerebral concentrations of KYNA[50,51]. It is now appreciated that cerebral 3-hydroxykynurenine and quinolinic acid concentrations can be manipulated, selectively without altering KYNA concentrations, by inhibition of KMO[49], an approach which shows neuroprotective effects in animal models of cerebral ischemia[19]. In addition, a newly synthesized compound, ZL006, which acts downstream of NMDA receptor activation, has been demonstrated to ameliorate focal ischemic cerebral damage induced by middle cerebral artery occlusion in mice and rats[52]. The implication of endogenous excitotoxicity in the present study suggests that ZL006 might also be evaluated to improve cognitive outcomes.

In the present study, the K/T ratio was able to identify subjects at risk of significant PSCI (i.e. those with sMMSE scores ≤ 24). While larger studies would be needed to confirm a clinically important cut-off value for the K/T ratio, the ROC presented in the present study suggested a 67% sensitivity and 86% specificity for a K/T ratio cut-off of 78.3 μM/mM. While higher K/T ratios were associated with lower MMSE scores in the entire population, this effect was most significant for subjects with a K/T ratio in the highest tertile. A K/T ratio in this range is comparable to that found in subjects with HIV-1 infection in association with cognitive symptoms and AIDS dementia complex[53-55], but much higher than that found in normal healthy aged controls[15] or subjects with cardiovascular disease without a history of stroke[43]. If replicated in larger populations, these data suggest potential clinical utility of the K/T ratio as a discriminating biomarker.

This study demonstrates a relationship between cognitive impairment and activation of the kynurenine pathway post-stroke. It is, however, limited by its use of a brief cognitive screening instrument and a relatively small sample size. Although stroke severity was assessed and included as a covariate, the timing of administration of the NIHSS relative to the stroke and cognitive assessment varied between assessments. Because NIHSS scores commonly fluctuate post-stroke, this measure may not have been adequate to control for stroke severity. However, in recognition of this, we also evaluated lesion volume and found that it was not significantly related to the severity of PSCI in this population. Additionally, the type of cognitive impairment observed post-stroke varies; often including deficits in measures of global cognition, as well as domain-specific impairments involving executive function, language, visuospatial ability, and memory[56]. Some previously identified risk factors for the development of PSCI, including family history of dementia and individual cardiovascular risk factors[8], could not be controlled for given the sample size. Although all efforts were made to control for potentially confounding variables, pre-stroke cognitive capacity and white matter disease burden were not assessed. Finally, the cross-sectional design of this study was useful in establishing an initial relationship between PSCI and elevated K/T ratios post-stroke, but subsequent longitudinal studies will be necessary to determine the value of K/T ratio in predicting long-term cognitive outcomes.

## Conclusions

In summary, an acute inflammatory response characterized by IDO activation may be relevant to the development of PSCI. Since the neuroactivities of kynurenine metabolites may be amenable to pharmacotherapeutic intervention, the K/T ratio may be a clinically important biomarker. Longitudinal observational and intervention studies will improve our understanding of the role of IDO activity in PSCI.

## Competing interests

The authors declare that they have no competing interests with respect to the authorship and/or publication of the article.

## Authors' contributions

This study is based on the original idea of KLL and NH. ABG participated in coordination and ABG and WS performed the statistical analysis and wrote the manuscript. KLL, NH, SEB, GT, and AK made contributions to conception and design and analysis and interpretation of data. RIA provided neuroimaging data. All authors read and approved the final manuscript.
